# Engagement in Particulate Matter Exposure Reduction Behaviors Across Diverse Clinical Cohorts

**DOI:** 10.3390/medicina62040689

**Published:** 2026-04-03

**Authors:** Sung Woo Moon, Ji Ye Jung, Hyun Woo Ji, Dae-Ryong Kang, Yong Jin Lee, Jin-Bae Kim, Yeong-Bae Lee, Changsoo Kim, Jaelim Cho, Mi-Ji Kim, Hye Jin Park, Young Sam Kim

**Affiliations:** 1 Division of Pulmonary and Allergy, Department of Internal Medicine, Konkuk University Medical Center, Konkuk University School of Medicine, Seoul 05030, Republic of Korea; 2 Division of Pulmonary and Critical Care Medicine, Department of Internal Medicine, Institute of Chest Diseases, Severance Hospital, Yonsei University College of Medicine, Seoul 03722, Republic of Korea; 3 Division of Pulmonology and Allergy, Department of Internal Medicine, National Health Insurance Service Ilsan Hospital, Goyang 10444, Republic of Korea; 4 Department of Precision Medicine, Yonsei University Wonju College of Medicine, Wonju 26426, Republic of Korea; 5 Institute of Environmental Research, College of Medicine, Yonsei University, Seoul 03722, Republic of Korea; 6 Division of Cardiology, Department of Internal Medicine, Kyung Hee University Hospital, Kyung Hee University, Seoul 02447, Republic of Korea; 7 Department of Neurology, Gachon University Gil Medical Center, Gachon University College of Medicine, Incheon 21565, Republic of Korea; 8 Department of Preventive Medicine, Yonsei University College of Medicine, Seoul 03722, Republic of Korea; 9 Department of Preventive Medicine and Institute of Health Science, Gyeongsang National University College of Medicine, Jinju 52727, Republic of Korea

**Keywords:** particulate matter, chronic obstructive pulmonary disease, atrial fibrillation, cerebrovascular, asthma, stroke

## Abstract

*Background and Objectives*: We aimed to evaluate differences in PM exposure reduction behaviors among older adults and patients with chronic respiratory diseases, atrial fibrillation, and stroke and to identify associated determinants. *Materials and Methods*: This multicenter cross-sectional study included 717 participants from five cohorts: older adults (*n* = 255), chronic obstructive pulmonary disease (COPD, *n* = 145), asthma (*n* = 100), atrial fibrillation (*n* = 117), and stroke (*n* = 100). PM exposure reduction behaviors were assessed using a standardized 18-item questionnaire (total score range: 0–126) covering indoor, outdoor, and other behaviors (health-seeking actions, such as checking air quality and using purifiers). *Results*: In multivariable linear regression models, COPD (β = 11.09), asthma (β = 15.54), and atrial fibrillation (β = 14.29) were associated with significantly higher total behavior scores compared with the older adult cohort (adjusted mean 69.0), corresponding to an approximately 20% relative increase in the asthma cohort. The stroke cohort did not differ significantly from the primary cohort. Domain-specific analyses showed that indoor and other behavioral scores were consistently higher across all disease cohorts, whereas outdoor scores were significantly elevated only for asthma and atrial fibrillation. In a fully adjusted model that included all covariates simultaneously, the stroke cohort demonstrated a modest increase in the total score (β = 8.79). Increased age and higher educational attainment were independently associated with greater behavioral engagement. *Conclusions*: PM exposure reduction behavior scores differed across the clinical cohorts, and sociodemographic factors were independently associated with behavioral engagement. These findings support personalized disease-specific counseling and inform future digital health interventions for vulnerable populations.

## 1. Introduction

Particulate matter (PM) exposure has been consistently associated with adverse respiratory, cardiovascular, and cerebrovascular health outcomes [1,2,3]. Older adults and individuals with chronic respiratory or cardiovascular diseases are generally considered vulnerable populations with increased susceptibility to air pollution-related health effects [4]. Although substantial research has examined the relationship between PM exposure and clinical outcomes [5,6], comparatively less attention has been directed toward understanding how vulnerable clinical populations engage in behaviors intended to reduce exposure [7,8].

Efforts to improve ambient air quality are ongoing; however, environmental- and policy-level interventions typically require long-term implementation [9]. In parallel, individual-level exposure reduction behaviors may represent a complementary strategy to mitigate potential health risks [10]. Such behaviors include indoor environmental management (e.g., use of air purifiers and ventilation behaviors), outdoor exposure modification (e.g., limiting outdoor activity during periods of elevated pollution or using protective masks), and other related behaviors such as monitoring air quality information. The extent to which these behaviors are adopted may vary according to clinical status, demographic characteristics, socioeconomic factors, and residential environment [11].

Patients with chronic respiratory diseases frequently receive counseling regarding environmental triggers [12], and individuals with cardiovascular conditions may be advised to avoid environmental stressors [2]. These clinical interactions could potentially influence engagement in exposure reduction behaviors [13]. In contrast, older adults without specific cardiopulmonary diagnoses may exhibit different behavioral patterns; they serve as a crucial baseline reference because they share age-related physiological decline and a generalized vulnerability to air pollution, but without the acute healthcare engagement characteristic of chronic disease cohorts [9]. Patients with cerebrovascular disease represent another clinically relevant population that is highly susceptible to recurrent events due to PM-induced systemic inflammation, endothelial dysfunction, and increased sympathetic tone [14,15]. However, direct comparisons of exposure reduction behaviors across respiratory, cardiovascular, stroke, and older adult cohorts remain limited.

Previous behavioral studies have often focused on single disease groups or the general population, which restricts their ability to directly compare heterogeneous clinical cohorts within a unified analytical framework [16,17]. Comparing these diverse clinical groups is essential to identify disease-specific gaps in environmental health literacy, as behavioral adaptation may vary significantly depending on the nature of the disease and specific medical counseling received. As a result, it remains unclear whether engagement in PM exposure reduction behaviors differs across distinct vulnerable clinical populations when evaluated under comparable conditions and adjusted for relevant covariates.

To address this gap, we conducted a multicenter cross-sectional study that included cohorts of older adults and individuals with chronic obstructive pulmonary disease (COPD), asthma, atrial fibrillation, and stroke. We compared the total and domain-specific PM exposure reduction behavior scores across these cohorts using a standardized questionnaire. In addition, we examined the demographic, socioeconomic, and environmental factors associated with this behavior. By characterizing the patterns of exposure reduction behaviors within and across clinically vulnerable populations, our findings provide objective evidence that may inform future research and targeted intervention strategies. Consistent PM avoidance acts as a crucial non-pharmacological intervention, reducing cumulative exposure and mitigating exacerbation or recurrence risks [18,19]. Identifying behavioral gaps across these specific cohorts underscores the clinical and policy necessity of expanding targeted, disease-specific environmental education [20].

## 2. Materials and Methods

### 2.1. Study Participants and Design

This study was conducted as part of the Environmental Health Digital Project and supported by the Korea Environmental Industry & Technology Institute and the Ministry of Environment in 2021. This government-funded, multicenter initiative leverages digital health technologies to monitor longitudinal environmental exposure and clinical parameters in vulnerable groups. Its primary objective is to implement personalized risk assessments and protective behavioral interventions using AI-based predictive models, specifically targeting PM-sensitive populations such as those with chronic respiratory or cardiovascular conditions. The primary objective of this study was to establish a living laboratory to develop personalized environmental health service models for vulnerable populations by monitoring real-time vital signs, clinical symptoms, and personal exposure to environmental hazards. Participants were recruited from multicenter clinical cohorts established at five tertiary university institutions. For the present cross-sectional analysis, we analyzed data from participants specifically recruited during the first year of the project in 2021. From the diverse original cohorts (which included children and industrial residents), we strategically selected five adult groups: older adults, chronic obstructive pulmonary disease (COPD), asthma, atrial fibrillation, and stroke. The four disease cohorts were chosen to represent major chronic cardiopulmonary and cerebrovascular conditions with established physiological susceptibility to particulate matter, allowing us to evaluate the impact of clinical vulnerability on PM exposure reduction behaviors. The older adult cohort served as an age-appropriate reference group. Other original cohorts were excluded to maintain a focused, consistent comparison of adult clinical populations.

The specific inclusion criteria for each cohort were as follows: (1) COPD: Patients aged ≥40 years with airflow limitation, defined as a forced expiratory volume in 1 s to forced vital capacity (FEV1/FVC) ratio < 0.7 (post-bronchodilator) on spirometry according to the GOLD criteria; (2) Asthma: Patients with a physician-confirmed diagnosis based on GINA guidelines and clinical symptoms who were receiving inhaled medications; (3) Atrial Fibrillation: Adults aged ≥19 years who provided informed consent and had a current or prior documented diagnosis via electrocardiography (ECG) at the cardiology clinic or had undergone implantation of a cardiac electrical device (including a pacemaker, implantable cardioverter-defibrillator, or implantable loop recorder); (4) Stroke: Patients aged ≥40 years with a history of chronic ischemic or hemorrhagic stroke confirmed by brain imaging (CT or MRI); (5) Older Adults: Community-dwelling individuals aged ≥65 years without a specific cardiopulmonary diagnosis, selected as the reference group for comparisons with disease-specific vulnerable cohorts. A total of 717 participants were included in the final analysis: older adults (*n* = 255); COPD (*n* = 145); asthma (*n* = 100); atrial fibrillation (*n* = 117); and stroke (*n* = 100).

All participants completed a standardized questionnaire assessing their daily PM exposure reduction behaviors aimed at reducing PM exposure to particulate matter. Demographic, socioeconomic, and residential information were collected concurrently. This study was designed as a multicenter cross-sectional analysis to compare PM exposure reduction behaviors across heterogeneous clinical cohorts rather than to evaluate longitudinal outcomes.

The following data were collected using standardized questionnaires and structured forms: demographic characteristics (age and sex); anthropometric measures (body mass index [BMI]); smoking history (ever-smoking status and pack-years); socioeconomic characteristics (educational attainment and household income); residential environment (housing type, heating fuel type, and distance from residence to the main road); lifestyle-related variables (outdoor activity time in hours/day); and physician-diagnosed comorbidities, including hypertension, diabetes mellitus, dyslipidemia, ischemic heart disease, stroke, arthritis, cancer, and psychiatric disorders.

Comorbidity data were collected from cohort-specific clinical records and questionnaires. Due to differences in data collection protocols across subcohorts, comorbidity information was unavailable for a subset of participants in certain cohorts.

Continuous variables included age, BMI, smoking exposure (pack-years), and time spent outdoors (hours/day). The categorical variables included sex, smoking status, educational level, household income, housing type, heating fuel type, residential distance from the main road, and comorbidities.

### 2.2. Scoring PM Exposure-Reduction Behaviors

PM exposure reduction behaviors were assessed using a structured questionnaire consisting of 18 items covering indoor behaviors (e.g., using air purifiers and regular ventilation), outdoor behaviors (e.g., limiting outdoor activities and avoiding high-traffic or industrial areas), and other health-related behaviors (e.g., checking air quality forecasts, drinking plenty of water, and consuming fruits and vegetables) (Supplementary Table S1). Participants were asked to report how frequently they performed each behavior during the past 12 months when the air quality was reported as “bad” or “very bad.” Each item was rated on an 8-point Likert scale ranging from 0 (never) to 7 (always). Higher scores indicated more frequent engagement in the specified behavior. To ensure a standardized comparison, disease-specific items (e.g., smoking and inhaler use) that were not universally measured across all five cohorts were excluded from the final scoring. These items were retained in the original questionnaire but were not included in the calculation of domain-specific or total behavior scores in this study. Therefore, the total behavior score was calculated as the sum of the remaining items applicable to all participants. Domain-specific scores (indoor, outdoor, and other behaviors) were computed by summing the corresponding items within each domain. All included items were weighted equally.

PM exposure reduction behaviors were assessed using a structured 18-item questionnaire developed within the “Environmental Health Digital Project” framework. To ensure content validity, the items were reviewed and refined by a panel of clinical experts. For the present analysis, items applicable only to specific subgroups (e.g., smoking and inhaler use) were excluded to ensure methodological standardization across cohorts. Consequently, the final behavior score was calculated based on 18 shared items, comprising six items for indoor, eight for outdoor, and four for other behaviors. Each item was rated on an 8-point Likert scale (0–7), resulting in a total score range of 0–126, with higher scores indicating more frequent engagement. The instrument demonstrated robust reliability, with an overall Cronbach’s alpha of 0.814 (sub-domains: indoor 0.723, outdoor 0.697, and other 0.586). These results imply that the instrument is a reliable tool for assessing environmental health engagement across diverse clinical groups.

### 2.3. Statistical Analysis

Baseline characteristics were compared across the five clinical cohorts using one-way analysis of variance for continuous variables and the chi-square test for categorical variables. Continuous variables are presented as mean ± standard deviation and categorical variables as numbers (percentages). No formal hypothesis testing was performed for the baseline comparisons. Comorbidity data were collected from cohort-specific clinical records and questionnaires. Because the data collection protocols differed across the subcohorts, comorbidity information was unavailable for a subset of participants in certain groups. Accordingly, descriptive summaries of the comorbid conditions were calculated using the data available within each cohort.

Unadjusted total and domain-specific PM exposure reduction behavior scores were calculated as cohort-level means and are presented graphically. Multivariable linear regression models were constructed using the older adult cohort as the reference group to assess differences in behavioral scores across clinical cohorts while accounting for potential confounders. Separate models were fitted for total and domain-specific scores (indoor, outdoor, and other behaviors), adjusting for age, sex, smoking exposure (pack-years), BMI, education level, household income, distance from residence to the main road (<100 m vs. ≥100 m), and outdoor activity time (hours/day). Regression coefficients (β) with 95% confidence intervals (CIs) are reported, and adjusted marginal means were derived from these models.

Additional multivariable analyses were performed to identify factors independently associated with the total behavioral score in the overall population and in disease-specific comparisons. Comorbidity variables were not included in the primary multivariable models because of their incomplete and heterogeneous availability across cohorts, which could compromise comparability. Model assumptions were evaluated using residual diagnostics and multicollinearity was assessed using variance inflation factors. Missing data were handled via complete case analysis; participants with missing covariates were excluded from regression models, while Table 1 descriptive statistics utilized all available data. A post hoc power analysis confirmed the adequacy of our sample size. With a total of 717 participants and an alpha level of 0.05, the study provided >95% statistical power to detect an effect size (f^2^) of 0.04 in the multivariable linear regression models. For the primary comparisons between disease cohorts and older adults, the study achieved a power of >99% to detect the observed effect sizes (Cohen’s *d* > 0.52), ensuring the robustness of our main findings. Statistical analyses were conducted using R (version 4.1.3; R Foundation for Statistical Computing, Vienna, Austria), with two-sided *p*-values < 0.05 considered statistically significant.

## 3. Results

### 3.1. Baseline Characteristics of the Study Population

The baseline characteristics of the study population are presented in Table 1. The COPD cohort had the highest proportion of men (87%), whereas 45% of the older adult cohort were men. The mean age was highest in the COPD group (69 ± 8 years) and lowest in the asthma group (49 ± 15 years). Outdoor activity time differed across cohorts, with the older adult group reporting the longest duration (3.07 ± 3.11 h/day), while the atrial fibrillation group reported the shortest (0.83 ± 1.65 h/day). Educational attainment varied substantially: 49% of the older adult cohort had completed high school or higher education, compared with 70% in the COPD cohort, 82% in the asthma cohort, 80% in the atrial fibrillation cohort, and 63% in the stroke cohort. Differences were also observed in household income distribution and housing characteristics across the groups. Detailed item-level mean scores for all 18 behaviors across the cohorts are provided in Supplementary Figure S1, highlighting specific areas of high engagement such as mask-wearing and air quality monitoring.

### 3.2. Comparison of PM Exposure Reduction Behavior Scores Across Cohorts

**Figure 1:**
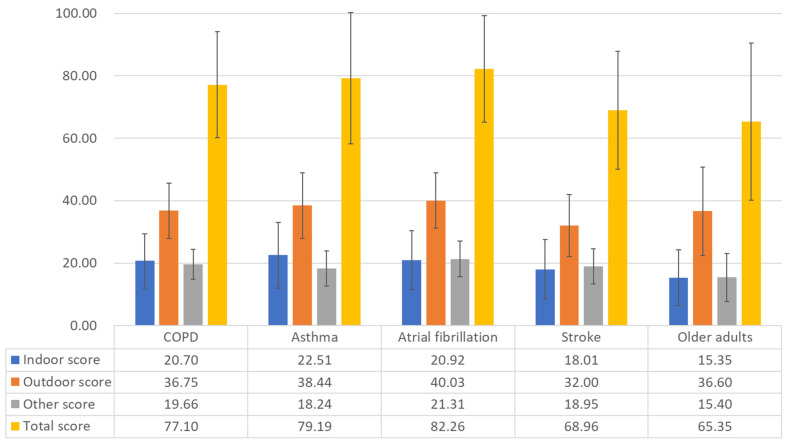
Comparison of unadjusted mean PM exposure reduction behavior scores by study cohort. The bar chart presents the raw (unadjusted) mean scores for indoor, outdoor, other, and total PM exposure reduction behaviors in each cohort. Error bars represent standard deviations (SD).

Unadjusted comparisons demonstrated that the total PM exposure reduction behavior scores were lowest in the older adult cohort and higher in the disease cohorts, particularly in the asthma and atrial fibrillation cohorts (Figure 1 and Figure S1). Indoor behavior scores showed more pronounced differences between the cohorts, whereas outdoor behavior scores were relatively similar across groups.

**Figure 2:**
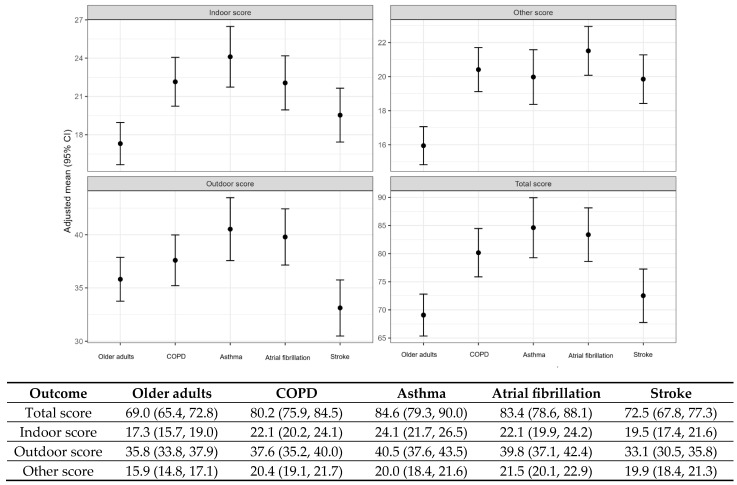
Adjusted marginal means and 95% confidence intervals of PM exposure reduction behavior scores across disease groups. Adjusted marginal means and 95% confidence intervals were estimated using multivariable linear regression models adjusted for age, sex, smoking status, body mass index, education level, household income, distance from residence to the main road, and outdoor activity time.

To evaluate behavioral differences across clinical groups, multivariable linear regression models were constructed using the older adult cohort as a reference (Table 2). After adjusting for demographic and lifestyle factors (age, sex, smoking status, BMI, educational attainment, household income, distance to the main road, and outdoor activity time), participants with COPD (β = 11.09, 95% CI 6.39–15.78, *p* < 0.001), asthma (β = 15.54, 95% CI 9.95–21.13, *p* < 0.001), and atrial fibrillation (β = 14.29, 95% CI 8.87–19.72, *p* < 0.001) demonstrated significantly higher total behavior scores compared with the older adult cohort. The stroke cohort, however, did not show a statistically significant difference in the total score (β = 3.44, 95% CI −1.51–8.39, *p* = 0.173). The adjusted marginal means (Figure 2) confirmed these cohort-level differences. Beyond statistical significance, the magnitude of these differences was assessed using Cohen’s *d*. The asthma and atrial fibrillation cohorts showed medium-to-large effect sizes in total behavioral engagement, while the COPD cohort exhibited a medium effect size. In contrast, the stroke cohort demonstrated a small effect size, indicating that the clinical impact on overall PM-protective behaviors was relatively limited in this group.

Domain-specific analyses revealed distinct behavioral patterns. Indoor and “other” behavioral scores were significantly higher in all disease cohorts than in the older adult cohort. In contrast, the outdoor behavior scores were significantly elevated only in the asthma and atrial fibrillation cohorts, whereas the COPD cohort showed no significant difference, and the stroke cohort exhibited a borderline reduction.

The adjusted marginal means derived from the multivariable linear regression models confirmed these differences (Figure 2). Domain-specific analyses adjusted for age, sex, smoking exposure, body mass index, education level, household income, residential distance to the main road, and outdoor activity time showed that indoor scores were significantly higher in all disease cohorts than in the older adult cohort. Outdoor scores were significantly higher in the asthma and atrial fibrillation cohorts, but not in the COPD cohort, while the stroke cohort showed a lower score. Other behavioral scores were significantly higher in all disease cohorts.

### 3.3. Factors Associated with Total PM Exposure Reduction Behavior Scores

To identify the independent determinants of the total PM exposure reduction behavior scores across the entire study population, a fully adjusted multivariable linear regression model was constructed that simultaneously incorporated all clinical, demographic, socioeconomic, and residential variables (Table 3).

In this fully adjusted model, the associations between the clinical cohorts and higher behavioral scores remained robust. Notably, after accounting for all covariates including residential characteristics, the stroke cohort demonstrated a modest but statistically significant increase in the total score compared with the older adult cohort (β = 8.79, 95% CI 0.67–16.91, *p* = 0.034), alongside the significantly higher scores maintained by the COPD, asthma, and atrial fibrillation cohorts.

Beyond clinical diagnosis, several sociodemographic and environmental factors emerged as independent associations with behavioral engagement. Higher age (β = 0.54 per year, 95% CI 0.30–0.78, *p* < 0.001) and higher educational attainment (high school or higher; β = 7.45, 95% CI 2.58–12.32, *p* = 0.003) were positively associated with the total score. Regarding residential environments, participants using oil heating (β = 12.33, 95% CI 4.49–20.17, *p* = 0.002) or “other” heating fuels (β = 8.59, 95% CI 0.68–16.50, *p* = 0.033) exhibited significantly higher scores compared to those using gas heating. In contrast, smoking exposure, household income, and housing type were not independently associated with the total score after full adjustment.

Disease-specific analyses demonstrated that higher educational attainment remained positively associated with the total score across all clinical cohorts when compared with the older adult cohort. The lower behavioral scores in the older adult cohort may be attributed to their distinct socioeconomic and residential profiles. Specifically, a higher prevalence of detached housing (65%), limited use of gas heating (30%), and lower educational attainment (49% high school graduates) in this group may act as structural and health literacy barriers to implementing effective PM reduction measures. Smoking burden continued to demonstrate negative associations in several cohort-specific models.

In summary, particulate matter exposure reduction behaviors varied significantly across clinical cohorts. While patients with asthma, atrial fibrillation, and COPD demonstrated robust engagement characterized by medium-to-large effect sizes, the stroke cohort exhibited a unique and less pronounced behavioral profile compared with older adults. These disparities were further influenced by sociodemographic determinants, specifically age and educational attainment, highlighting that behavioral engagement is driven by both clinical diagnosis and individual background factors.

## 4. Discussion

In this multicenter cross-sectional study integrating diverse clinical cohorts, we observed differential levels of engagement in PM exposure reduction behaviors among older adults and patients with COPD, atrial fibrillation, and stroke. After adjusting for demographic, socioeconomic, and residential factors, the COPD, asthma, and atrial fibrillation cohorts demonstrated significantly higher total behavioral scores than the older adult cohort, whereas the stroke cohort did not show a statistically significant difference in the overall score. Domain-specific analyses indicated that differences were most pronounced in indoor and “other” behavior domains, while outdoor behaviors showed smaller or inconsistent between-group differences.

Given that the total behavior score ranged from 0 to 126, the approximately 16-point higher adjusted score in the asthma cohort compared to the older adult cohort (mean 69.0) represented a 23% relative increase and nearly one category shift per item. This difference suggests a meaningful divergence in day-to-day engagement with PM exposure reduction practices.

Prior research has typically examined behaviors within isolated clinical groups or the general population, without directly comparing diverse vulnerable cohorts under standardized conditions. Although some surveys have included multiple subgroups, direct comparisons of diverse clinically vulnerable cohorts under standardized assessment conditions remain limited. In COPD and asthma, behavioral research has largely focused on individual-level protective actions, such as monitoring air quality, reducing outdoor activity during high-PM episodes, and managing indoor air environments, often within targeted educational or interventional settings [8,9]. In cardiovascular disease populations, professional societies recommend minimizing exposure during high-pollution periods [10,21]; however, real-world data on actual behavioral engagement remain limited. For stroke and other cerebrovascular diseases, the literature has primarily examined exposure–outcome associations, with relatively little attention paid to patient-level exposure-reduction behaviors [22,23]. Among older adults, studies more commonly report reduced outdoor activity rather than structured environmental management behaviors [24]. The present study addresses this gap by assessing PM exposure reduction behaviors across multiple clinical populations within a unified analytical framework with multivariable adjustment.

In contrast, the stroke cohort did not demonstrate a statistically significant increase in the total behavioral score compared with the older adult cohort after adjustment. This may reflect differences in disease perception, rehabilitation priorities, and functional limitations that affect behavioral choices. Specifically, the intensive focus on neurological rehabilitation may prioritize these clinical goals over environmental PM exposure reduction behaviors. Additionally, post-stroke physical or cognitive impairments could pose practical barriers to consistently performing active protective measures, such as mask use or air filter maintenance. However, these interpretations remain speculative as this study did not evaluate awareness, cognitive status, or physical function. Notably, indoor and other behavioral domains were elevated in the stroke group despite the lack of a significant difference in the total score, suggesting that engagement patterns may vary across behavioral categories rather than uniformly across all behaviors. In the full multivariable model, which included all covariates simultaneously (Table 3), the association in the stroke cohort reached statistical significance, although the magnitude of the effect remained modest. This pattern suggests that the estimated association may be sensitive to the model specification and adjustment strategy.

Several sociodemographic factors were independently associated with total behavioral scores. Higher educational attainment was consistently associated with greater engagement, whereas greater smoking was associated with lower scores. Increasing age showed a positive association with the scores after adjustment. These findings indicate that behavioral engagement is influenced by demographic and lifestyle characteristics in addition to clinical diagnosis. Socio-demographic factors significantly shape these behavioral responses. Higher educational attainment likely reflects greater environmental health literacy, facilitating the comprehension of complex air quality data. Conversely, residential factors—such as housing type and heating fuel—represent structural and financial barriers that limit environmental modifications. Therefore, effective public health strategies must move beyond general information provision to integrate tailored education with practical socioeconomic support.

Behavioral responses to environmental risks are shaped not only by clinical vulnerability, but also by risk perception, social context, and individual adaptive capacity [25,26]. Within this broader framework, the modest inverse association observed between high household income and total behavior score should be interpreted cautiously. This association may reflect unmeasured contextual factors or cohort composition and does not imply reduced preventive capacity in higher-income groups.

These domain-specific results provided additional insights. Indoor behaviors showed the most consistent differences across cohorts, possibly reflecting that indoor environmental management is more feasible and controllable, particularly for individuals with limited mobility. In contrast, outdoor behaviors demonstrated greater heterogeneity; they were significantly elevated in the asthma and atrial fibrillation cohorts but not in the COPD cohort and showed a borderline reduction in the stroke cohort. Outdoor PM exposure reduction behaviors require active mobility and timely adaptation to air quality conditions [27]. Patients with asthma may be more responsive to acute pollution-related symptom triggers, whereas individuals with COPD or stroke may have baseline physical or functional limitations that constrain outdoor behavioral modification. These findings suggest that outdoor PM exposure reduction behaviors may depend not only on the disease category but also on symptom perception and functional capacity.

The observed behavioral differences among the clinical cohorts may also be driven by their distinct clinical contexts. Higher scores in respiratory patients likely reflect the positive impact of routine environmental counseling typically provided in standard pulmonary care. In contrast, the lower engagement seen in stroke and older adult cohorts may be attributed to physical or cognitive barriers that hinder active exposure reduction measures. Additionally, underlying psychological conditions, such as depression, common across chronic diseases, can broadly diminish proactive PM exposure reduction behaviors. Finally, the 2021 study period during the COVID-19 pandemic likely inflated absolute behavioral scores across all cohorts due to universal mask mandates and heightened respiratory vigilance [28]. Nevertheless, the relative differences observed between clinical groups remain informative, as all cohorts were evaluated under identical pandemic-related public health conditions.

Our findings advocate personalized environmental health counseling over generic messaging, emphasizing the integration of disease-specific PM reduction strategies into clinical guidelines. The lower engagement observed in the stroke cohort presents a critical opportunity for non-respiratory specialists (e.g., neurologists and cardiologists) to prioritize targeted patient education. These results provide an empirical foundation for developing digital health interventions, informing the design of real-time, personalized alert systems tailored to the behavioral patterns of vulnerable clinical populations.

This study has several strengths. First, it directly compared heterogeneous clinical cohorts, including those with respiratory diseases, atrial fibrillation, stroke, and older adults within a unified analytical framework using a standardized questionnaire. Second, multivariable adjustment was performed to account for demographic, socioeconomic, and residential characteristics, thereby reducing potential confounding factors. Third, the analysis of domain-specific behavior scores allowed for a more nuanced evaluation of the behavioral patterns.

This study also had several limitations. First, the cross-sectional design precludes inferences regarding causality or directionality between clinical status and behavioral engagement and introduces potential reverse causality, as individuals with more severe symptoms might reactively adopt stricter PM reduction behaviors. Consequently, the temporal sequence between the onset of specific clinical conditions and subsequent behavioral modifications cannot be definitively determined. Future longitudinal studies are necessary to elucidate the causal and temporal dynamics of these associations. Second, although the 18-item questionnaire was developed by an expert panel to ensure content validity, all behavioral measures were self-reported and may have been subject to recall or social desirability bias. Furthermore, as noted in the methods, the ‘other’ behavior sub-domain demonstrated modest reliability (Cronbach’s α = 0.586), which should be taken into account when interpreting these specific results. Moreover, certain confounders such as environmental awareness or prior medical advice were not captured. Third, we did not measure actual PM exposure levels, lack objective environmental monitoring data such as individual-level average ambient PM concentrations or days exceeding World Health Organization limits, and did not assess specific indoor air quality determinants, such as cooking methods and mold. Therefore, we could not evaluate whether the reported behaviors translated into measurable reductions in exposure. Fourth, as the data were collected in 2021 during the COVID-19 pandemic, heightened public awareness of respiratory protection and widespread mask use may have influenced the reported behaviors, potentially limiting the generalizability of the findings to non-pandemic periods. Fifth, the atrial fibrillation cohort in our study represents a highly heterogeneous population, as atrial fibrillation may be found in “healthy” hearts, as well as in valvular, ischemic, or myopathic heart disease. Furthermore, our inclusion criteria broadly encompassed cardiac electrical device recipients based on the overarching project’s definition of cardiovascular vulnerability. Because the dataset did not specify the exact underlying indication for each device, a sensitivity analysis strictly excluding non-AF recipients was not feasible. Sixth, comorbidity data were disproportionately missing in the older adult cohort (40.8%) due to structural differences in data collection across sites; thus, we adjusted for age, sex, and socioeconomic status as surrogates for health status instead of individual comorbidities. Similarly, due to disparate clinical metrics, we could not adjust for disease severity in a unified model. Seventh, participants were recruited from clinical cohorts within the participating institutions, which may introduce selection bias by potentially overrepresenting patients with higher baseline healthcare engagement, and generalizability to broader community populations may be limited. Finally, we did not apply statistical corrections for multiple comparisons due to the study’s exploratory nature. Consequently, borderline significant findings, such as those in the stroke cohort, warrant cautious interpretation.

Despite these limitations, this study provides comparative evidence that engagement in PM exposure reduction behaviors differs across specific clinical populations. Higher engagement among individuals with COPD, asthma, and atrial fibrillation suggests that disease-specific factors may influence preventive behaviors. Sociodemographic characteristics, particularly educational attainment and tobacco smoking, were independently associated with behavioral engagement across cohorts. These findings advocate for personalized, disease-specific counseling involving non-respiratory specialists, establishing an empirical foundation for future digital health-based interventions to protect vulnerable populations from environmental hazards.

## 5. Conclusions

Engagement in PM exposure reduction behaviors varied across diverse clinical cohorts, with higher participation observed among patients with chronic respiratory diseases and atrial fibrillation than among older adults. In contrast, the stroke cohort exhibited a unique, less pronounced behavioral profile, likely reflecting specific clinical and functional constraints. Sociodemographic factors also played an independent role. These findings support an empirical foundation for future digital health-based monitoring and intervention systems designed to protect vulnerable populations. Further longitudinal studies incorporating objective exposure measures and behavioral determinants are warranted to better understand the implications of these findings.

## Figures and Tables

**Table 1:** Baseline characteristics of the study population by cohort.

Characteristics	Older Adults (*n* = 255)	COPD (*n* = 145)	Asthma (*n* = 100)	Atrial Fibrillation (*n* = 117)	Stroke (*n* = 100)	*p*-Value
Sex, male, n (%)	116 (45)	126 (87)	38 (38)	81 (69)	54 (54)	<0.001
Age, years	66 ± 8	69 ± 8	49 ± 15	64 ± 9	64 ± 11	0.005
Body mass index, kg/m^2^	24.8 ± 3.1	23.9 ± 3.0	24.5 ± 3.8	25.9 ± 3.1	25.0 ± 3.4	<0.001
Ever smoker, n (%)	97 (38.0)	120 (82.8)	36 (36.0)	52 (44.4)	53 (53)	<0.001
* Comorbidities, n (%)						
Ischemic heart disease	20 (9.5)	4 (6.5)	6 (11)	3 (23)	10 (10)	<0.001
Hypertension	110 (52)	31 (50)	25 (45)	8 (62)	80 (80)	<0.001
Dyslipidemia	76 (36)	8 (13)	20 (36)	7 (54)	86 (86)	<0.001
Diabetes mellitus	42 (20)	7 (11)	6 (11)	3 (23)	24 (24)	<0.001
Stroke	6 (2.8)	2 (3.2)	2 (3.6)	0 (0)	90 (90)	<0.001
Arthritis	57 (27)	5 (8.1)	3 (5.4)	1 (7.7)	0 (0)	<0.001
Cancer	31 (15)	10 (16)	3 (5.4)	0 (0)	2 (2.0)	<0.001
Psychotic disease	13 (6.2)	5 (8.1)	2 (3.6)	2 (15)	2 (2.0)	<0.001
Education level, n (%)						<0.001
Middle school or less	130 (51)	44 (30)	18 (18)	23 (20)	37 (37)	
High school or higher	125 (49)	101 (70)	82 (82)	94 (80)	63 (63)	
Household income, n (%)						<0.001
Low to middle income	156 (61)	91 (63)	73 (73)	103 (88)	55 (55)	
High income	99 (39)	54 (37)	27 (27)	14 (12)	45 (45)	
Outdoor stay time (hours/day)	3.07 ± 3.11	1.48 ± 2.03	1.24 ± 1.73	0.83 ± 1.65	1.22 ± 2.66	<0.001
Environmental Residential Factors						
Distance to road, n (%)						0.046
<100 m	154 (60)	70 (48)	61 (61)	65 (56)	47 (47)	
≥100 m	101 (40)	75 (52)	39 (39)	52 (44)	53 (53)	
Housing type, n (%)						<0.001
Detached house	167 (65)	30 (21)	28 (28)	36 (31)	20 (20)	
Multi-unit house	20 (7.8)	37 (26)	20 (20)	31 (26)	24 (24)	
Apartment	66 (26)	76 (52)	52 (52)	47 (40)	51 (51)	
Etc.	2 (0.8)	2 (1.4)	0 (0)	3 (2.6)	5 (5.0)	
Heating fuel, n (%)						<0.001
Gas	77 (30)	129 (89)	76 (76)	110 (94)	80 (80)	
Oil	119 (47)	4 (2.8)	12 (12)	0 (0)	10 (10)	
Electricity	12 (4.7)	3 (2.1)	7 (7.0)	6 (5.1)	1 (1.0)	
Etc.	47 (18)	9 (6.2)	5 (5.0)	1 (0.9)	9 (9.0)	

COPD, chronic obstructive pulmonary disease. Note: Continuous variables are presented as mean ± SD, and categorical variables are presented as n (%). *p*-values were calculated using one-way analysis of variance for continuous variables and the chi-square test for categorical variables. * Note on comorbidities: Data on underlying comorbidities were missing for a subset of participants because of heterogeneous data collection protocols across the subcohorts. The numbers of patients with missing comorbidity data in each cohort were as follows: older adults (*n* = 44), COPD (*n* = 83), asthma (*n* = 45), atrial fibrillation (*n* = 104), and stroke (*n* = 0). Therefore, the effective denominators used to calculate the comorbidity percentages were as follows: older adults (*n* = 211), COPD (*n* = 62), asthma (*n* = 55), atrial fibrillation (*n* = 13), and stroke (*n* = 100).

**Table 2:** Adjusted Associations Between Disease Groups and PM exposure reduction Behavior Scores.

Disease Group (vs. Older Adults)	Total β (95% CI) [Cohen’s *d*]	*p*-Value	Indoor β (95% CI) [Cohen’s *d*]	*p*-Value	Outdoor β (95% CI) [Cohen’s *d*]	*p*-Value	Other β (95% CI) [Cohen’s *d*]	*p*-Value
COPD	11.09 (6.39, 15.78) [0.52]	<0.001	4.84 (2.75, 6.93) [0.54]	<0.001	1.78 (−0.82, 4.39) [0.15]	0.179	4.46 (3.05, 5.87) [0.70]	<0.001
Asthma	15.54 (9.95, 21.13) [0.73]	<0.001	6.80 (4.32, 9.28) [0.75]	<0.001	4.71 (1.61, 7.81) [0.40]	0.003	4.03 (2.35, 5.71) [0.63]	<0.001
Atrial fibrillation	14.29 (8.87, 19.72) [0.67]	<0.001	4.75 (2.35, 7.16) [0.53]	<0.001	3.98 (0.97, 6.98) [0.34]	0.01	5.56 (3.93, 7.19) [0.87]	<0.001
Stroke	3.44 (−1.51, 8.39) [0.16]	0.173	2.23 (0.03, 4.43) [0.25]	0.047	−2.69 (−5.44, 0.05) [0.23]	0.054	3.90 (2.42, 5.39) [0.61]	<0.001

Values in square brackets represent Cohen’s *d* as a measure of effect size. Multivariable linear regression was adjusted for age, sex, smoking status, body mass index (BMI), education level, household income, distance from residence to the main road, and outdoor activity duration.

**Table 3:** Multivariable linear regression analysis of factors associated with total PM exposure reduction behavior score.

Variables	Coefficient (β)	95% CI	*p*-Value
Cohort (Ref: Older adults)			
COPD	16.21	9.29, 23.12	<0.001
Asthma	19.59	10.45, 28.74	<0.001
Atrial fibrillation	22.87	14.52, 31.21	<0.001
Stroke	8.79	0.67, 16.91	0.034
Age (years)	0.54	0.30, 0.78	<0.001
Sex: Female (Ref: Male)	1.51	−6.44, 9.46	0.709
Body mass index, kg/m^2^	−0.28	−0.98, 0.43	0.441
Ever smoker (Ref; Never smoker)	−0.07	−0.16, 0.03	0.156
Education: High school or higher (Ref: Middle school or less)	7.45	2.58, 12.32	0.003
Household income: High income (Ref: Not high)	−4.89	−11.64, 1.86	0.155
Outdoor stay time (hours/day)	−0.27	−1.19, 0.65	0.559
Environmental Residential Factors			
Distance to road ≥ 100 m (Ref: <100 m)	0.22	−4.18, 4.61	0.922
Housing type (Ref: Detached house)			
Multi-unit house	−2.08	−8.95, 4.79	0.552
Apartment	1.15	−4.68, 6.98	0.698
Etc.	4.84	−12.36, 22.05	0.580
Heating fuel (Ref: Gas)			
Oil	12.33	4.49, 20.17	0.002
Electricity	−7.91	−20.12, 4.31	0.204
Etc.	8.59	0.68, 16.50	0.033

Abbreviations: β, regression coefficient; CI, confidence interval. The dependent variable was the total PM exposure reduction behavior score (original scale). All the variables listed in the table were simultaneously entered into the multivariable model. Reference groups are shown in parentheses. The intercept for this model was 26.55 (95% CI: 0.49, 52.66, *p* = 0.046).

## Data Availability

The data presented in this study are available upon request from the corresponding authors. The data are not publicly available due to ethical restrictions.

## Supplementary Materials

The following supporting information can be downloaded at https://www.mdpi.com/article/10.3390/medicina62040689/s1, Figure S1: Mean scores for individual PM exposure-reduction behavior items across clinical cohorts. Table S1: Domain Classification and Scoring of PM Exposure-Reduction Behavior Survey.
