# Supplementary material for: Engagement in Particulate Matter Exposure Reduction Behaviors Across Diverse Clinical Cohorts
Source: Medicina (Kaunas). 2026 Apr 3;62(4):689. doi: 10.3390/medicina62040689 (PMC13118065; doi:10.3390/medicina62040689)
Supplement: Supplementary file 1 [file medicina-62-00689-s001.zip › medicina-4212330-supplementary.pdf]

Supplementary Table S1. Domain Classification and Scoring of PM Exposure–Reduction Behavior Survey

Domain	Behavior Items
Indoor Behavior	- Use air purifiers<br>- Regular ventilation<br>- Check air purifier filters<br>- Use kitchen ventilator<br>- Clean using wet mopping<br>- Spray water before mopping
Outdoor Behavior	- Limit outdoor activities<br>- Wear certified particulate respirator masks outdoors<br>- Avoid high-traffic or industrial areas<br>- Switch to indoor air circulation while driving<br>- Reduce intense outdoor activity<br>- Wear protective clothing outdoors<br>- Clean body after outdoor exposure<br>- Remove dust from clothes before entering home
Other Behavior	- Check air quality forecast<br>- Drink plenty of water<br>- Consume fruits and vegetables<br>- Avoid secondhand smoke

Participants were asked to indicate how frequently they performed each behavior during the past 12 months when air quality was reported as “bad” or “very bad.” Responses were recorded on an 8-point Likert scale ranging from 0 (Never) to 7 (Always).

Total Score: Calculated as the sum of all item scores. Higher total scores reflect greater overall engagement in PM exposure–reduction behaviors.

Supplementary Figure S1. Mean scores for individual PM exposure–reduction behavior items across clinical cohorts

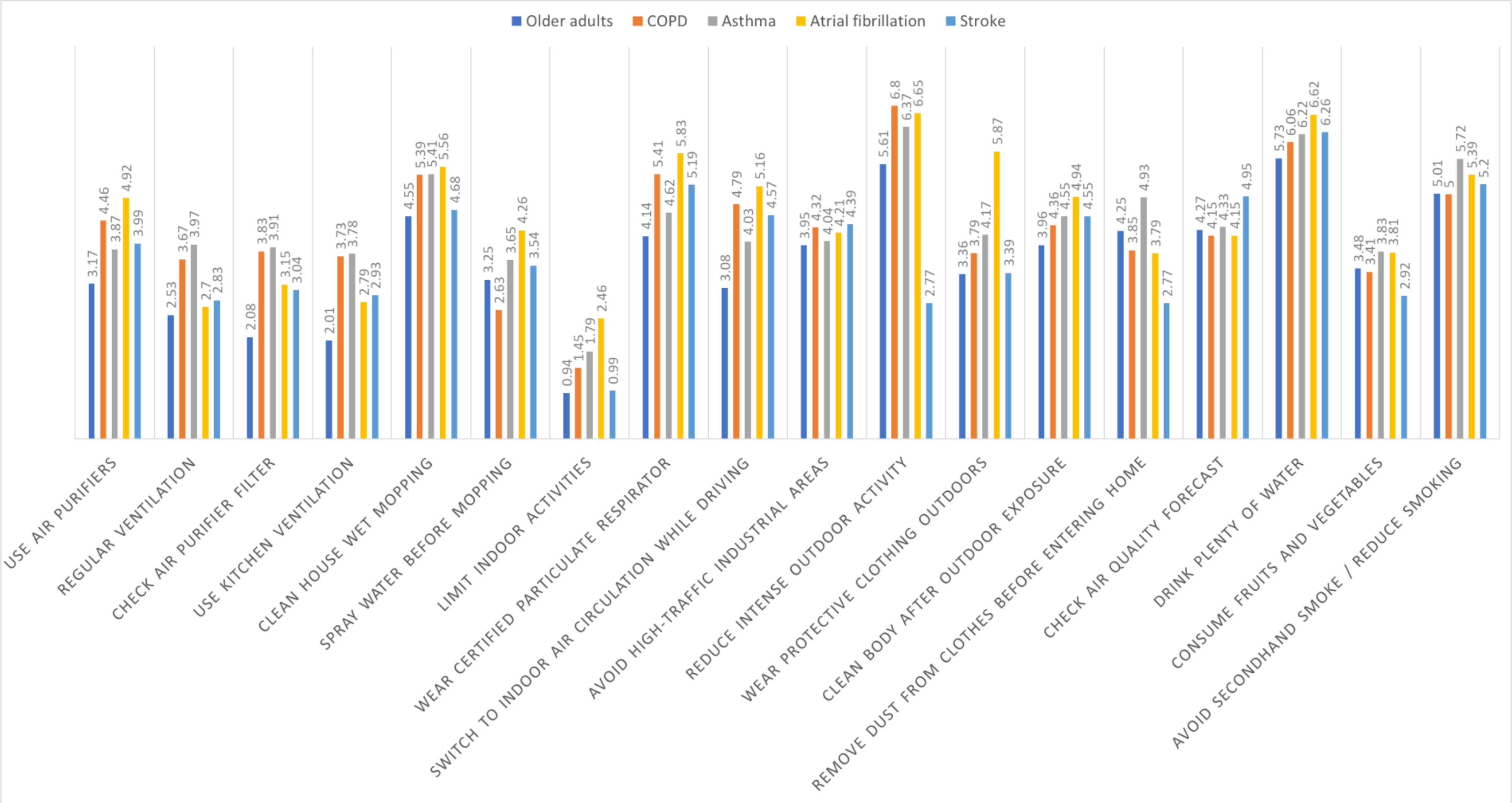

Bars represent mean item scores (0–7 Likert scale) for each cohort. Items are grouped by domain (Indoor, Outdoor, and Other behaviors).
